# Gut Dysbiosis and Microbiota-Derived Metabolites in Neurodegenerative Diseases: Molecular and Biochemical Mechanisms Along the Gut–Brain Axis

**DOI:** 10.3390/molecules31030490

**Published:** 2026-01-30

**Authors:** Patrycja Victoria Czaj, Karolina Szewczyk-Golec, Jarosław Nuszkiewicz, Alina Woźniak

**Affiliations:** 1 Student Research Club of Medical Biology and Biochemistry, Department of Medical Biology and Biochemistry, Faculty of Medicine, Ludwik Rydygier Collegium Medicum in Bydgoszcz, Nicolaus Copernicus University in Toruń, 85-092 Bydgoszcz, Poland; 310531@stud.umk.pl; 2 Department of Medical Biology and Biochemistry, Faculty of Medicine, Ludwik Rydygier Collegium Medicum in Bydgoszcz, Nicolaus Copernicus University in Toruń, 85-092 Bydgoszcz, Poland; karosz@cm.umk.pl

**Keywords:** Alzheimer’s disease, amyotrophic lateral sclerosis, gut–brain axis, gut microbiota, microbiota-derived metabolites, neurodegeneration, oxidative stress, Parkinson’s disease, short-chain fatty acids

## Abstract

Neurodegenerative disorders such as Alzheimer’s disease (AD), Parkinson’s disease (PD), and amyotrophic lateral sclerosis (ALS) share key molecular features, including neuroinflammation, oxidative stress, mitochondrial dysfunction, and progressive neuronal loss. Increasing evidence indicates that gut dysbiosis and alterations in microbiota-derived metabolites are involved in these processes through multiple pathways along the gut–brain axis. However, while broad compositional changes are well-documented, a critical knowledge gap remains regarding the specific biochemical signal transduction pathways translating dysbiosis into pathology. This narrative review addresses this gap by synthesizing current human and experimental studies addressing gut microbiota alterations in AD, PD, and ALS, with particular emphasis on the biochemical and molecular mechanisms mediated by gut-derived metabolites. Dysbiosis in neurodegenerative diseases is frequently associated with reduced abundance of short-chain fatty acid (SCFA)-producing bacteria and altered metabolism of SCFAs, bile acids, tryptophan-derived indoles, trimethylamine-N-oxide (TMAO), and lipopolysaccharides (LPS). These microbial metabolites have been shown to modulate intestinal and blood–brain barrier integrity, influence Toll-like receptor- and G protein-coupled receptor-dependent signaling, regulate microglial activation, and affect molecular pathways related to protein aggregation in experimental models. In addition, emerging evidence highlights the involvement of oxidative and nitrosative stress, immune–metabolic crosstalk, and altered xenobiotic metabolism in microbiota–host interactions during neurodegeneration. By integrating microbiological, metabolic, and molecular perspectives, this review underscores the important and emerging role of microbiota-derived molecules in neurodegenerative disorders and outlines key chemical and metabolic pathways that may represent targets for future mechanistic studies and therapeutic strategies.

## 1. Introduction

Neurodegenerative diseases (NDs), characterized by the progressive loss of structure and/or function of neurons, represent one of the most significant medical and social challenges of the 21st century. Despite extensive research, the exact etiopathogenesis of conditions such as Alzheimer’s disease (AD), Parkinson’s disease (PD), and amyotrophic lateral sclerosis (ALS) remains incompletely understood and is likely multifactorial, involving genetic, environmental, and metabolic components. Consequently, current therapeutic options are mostly symptomatic and fail to halt disease progression. Recent evidence has increasingly focused on the potential role of the gut microbiota (GM) as an important environmental factor in the development or exacerbation of NDs [1].

Although numerous studies have characterized GM composition in neurodegenerative diseases, changes in microbial taxonomy alone do not fully explain disease-related mechanisms. Increasing evidence suggests that microbiota-derived metabolites represent key functional mediators linking gut dysbiosis with neuroinflammation and neurodegeneration. Recent comprehensive reviews have significantly advanced our understanding of the microbiota–gut–brain axis in neurodegenerative disorders. For instance, Chen et al. [2] provided an extensive overview of immune and endocrine pathways, Loh et al. [3] highlighted the critical role of glial cell regulation and therapeutic interventions, and Missiego-Beltrán and Beltrán-Velasco [4] synthesized the general roles of broad metabolite classes, such as SCFAs and polyphenols. However, while these works admirably catalog microbiota composition changes and broad therapeutic strategies, a critical knowledge gap remains regarding the precise biochemical signal transduction pathways that translate microbial metabolic output into neurobiological effects. Unlike prior summaries, the present review distinguishes itself by focusing specifically on the molecular mechanisms of microbiota-derived metabolites—such as the receptor signaling of secondary bile acids and tryptophan derivatives—and bridges the gap between microbiological associations and neurochemical pathology.

The aim of this review is to synthesize current knowledge on microbiota-derived metabolites and their molecular and biochemical mechanisms along the gut–brain axis in Alzheimer’s disease, Parkinson’s disease, and amyotrophic lateral sclerosis.

### 1.1. The Gut Microbiota Ecosystem and Factors Influencing Its Composition

The human gastrointestinal tract harbors a complex and dynamic community of microorganisms. Bacterial density increases longitudinally along the tract, being lower in the stomach and proximal small intestine but rising dramatically in the large intestine to an estimated 10^11^–10^12^ bacteria per gram of colonic content, which contributes to approximately 60% of the fecal mass [5]. This delicate ecosystem is highly susceptible to external factors. Specific behaviors and medical interventions may directly influence the GM composition and diversity, including the use of antibiotics, proton pump inhibitors (antacids), anti-diabetic medications, or specific surgical procedures such as gastric bypass [6]. Furthermore, diet and nutrient intake are among the most potent modulators of GM, capable of shifting the microbial profile towards either a homeostatic or pro-inflammatory state [7].

Alterations in GM composition may result in profound changes in microbial metabolic output, including reduced production of short-chain fatty acids, altered bile acid profiles, and disrupted tryptophan metabolism.

### 1.2. The Microbiota–Gut–Brain Axis Pathways

**Figure 1:**
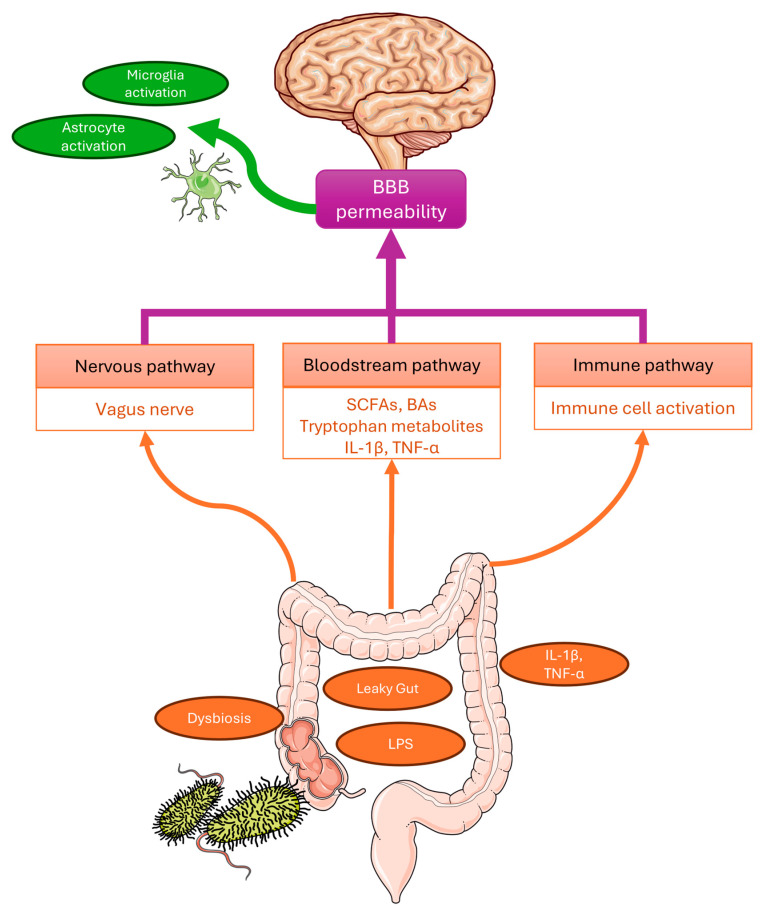
Schematic representation of the microbiota–gut–brain (MGB) axis communication pathways. Gut dysbiosis and increased intestinal permeability (“leaky gut”) facilitate the translocation of bacterial metabolites (e.g., lipopolysaccharides (LPS), short-chain fatty acids (SCFAs), bile acids (BAs)) and pro-inflammatory cytokines (interleukin-1β (IL-1β), tumor necrosis factor-α (TNF-α)) into the systemic circulation. Signaling to the central nervous system (CNS) occurs via three primary routes: (1) the neural pathway, primarily mediated by the vagus nerve; (2) the metabolic/bloodstream pathway, transporting microbiota-derived molecules that may influence blood–brain barrier (BBB) integrity; and (3) the immune pathway, involving cytokine signaling and immune cell activation. This figure highlights the systemic transport of molecular mediators that link gut dysbiosis with neuroinflammatory processes and microglial activation associated with neurodegenerative disorders. Images adapted from Servier Medical Art, licensed under CC BY 4.0.

It has been established that the gut and the central nervous system (CNS) are not isolated entities but are bidirectionally interconnected through the microbiota–gut–brain (MGB) axis. This communication occurs via three primary parallel pathways, including neural, immune, and endocrine/metabolic pathways (Figure 1).

The metabolic pathway involves specific neurotransmitters and neurotoxins produced or modulated by bacteria. These include short-chain fatty acids (SCFAs), bile acid derivatives (ligands for FXR and TGR5), acetylcholine, tryptophan metabolites (activators of the aryl hydrocarbon receptor, AhR), D-lactate, and ammonia [6]. SCFAs, among other things, signal through G-protein coupled receptors (e.g., GPR41, GPR43) to manage inflammation through mechanisms that include the control of neutrophil chemotaxis and the regulation of T regulatory cell proliferation [8,9]. Importantly, many of these microbial molecules can be transmitted through the systemic circulation and may influence blood–brain barrier (BBB) integrity, as well as indirectly modulate neural activity and microglial function. The neural pathway involves the direct connection between the enteric nervous system (ENS) and the CNS via the vagus nerve and the autonomic nervous system, allowing for rapid signal transmission [10]. The immune pathway facilitates interaction through the systemic circulation. The gut microbiome can influence the brain via the modulation of the immune system, here activation of Toll-like receptor 4 (TLR4) by bacterial lipopolysaccharides (LPS) triggers the release of pro-inflammatory cytokines, such as interleukin-1 (IL-1), interleukin-6 (IL-6), and tumor necrosis factor-α (TNF-α), thereby promoting neuroinflammatory processes [11].

When the homeostatic balance of the microbial community is disrupted—a state known as dysbiosis—the protective functions of the microbiota–gut–brain (MGB) axis may be compromised. AD, PD, and ALS all share the characteristic of distinct disturbances in the composition of the intestinal microbiota [12,13,14]. These alterations are increasingly recognized not merely as a consequence of the disease, but as a potential gateway to its pathogenesis and a novel target for prevention or treatment.

However, phylogenetic shifts alone do not fully explain the pathophysiology. To provide a comprehensive overview, this narrative review synthesizes current evidence on the functional consequences of dysbiosis. Specifically, it examines how alterations in microbial metabolism, ranging from the production of hydrophobic bile acids and neurotoxic kynurenines to the modulation of gamma-aminobutyric acid (GABA)ergic signaling via the vagus nerve, bridge the gap between the gut environment and neurodegeneration.

## 2. Key Microbial Metabolites Modulating Neurodegeneration

While phylogenetic shifts in microbiota composition are well-documented, the functional impact of dysbiosis is primarily mediated through alterations in specific metabolic profiles. Beyond classical inflammatory mediators, emerging evidence highlights the critical role of specific microbial metabolites including SCFAs, secondary bile acids, tryptophan metabolites and LPS in neurodegenerative pathology.

### 2.1. Short-Chain Fatty Acids: Epigenetic and Receptor-Mediated Signaling

Short-chain fatty acids (SCFAs), primarily acetate, propionate, and butyrate, are the major end-products of bacterial fermentation of dietary fibers. These metabolites exert their biological effects via two distinct but complementary molecular mechanisms: the activation of G-protein-coupled receptors (GPCRs) and the modulation of gene expression through epigenetic modifications. Maslowski et al. [9] identified GPR43 as a critical molecular link between the metabolic output of the GM and host immune regulation. Their study demonstrated that acetate and propionate binding to GPR43 on neutrophils and eosinophils is necessary for the resolution of inflammatory responses. Importantly, GPR43-deficient (Gpr43^−/−^) mice exhibited exacerbated inflammation in models of colitis and arthritis, characterized by increased recruitment of immune cells and elevated production of inflammatory mediators. This suggests that SCFA-GPR43 signaling serves as a critical negative regulator of immune activation, thereby limiting systemic inflammation that could otherwise compromise the CNS.

Epigenetic Regulation: Beyond receptor signaling, butyrate functions as a potent histone deacetylase (HDAC) inhibitor. This activity leads to increased histone acetylation, relaxing chromatin structure, and facilitating gene transcription. Furusawa et al. [8] demonstrated the precise molecular mechanism underlying this effect. They showed that butyrate induces the differentiation of colonic regulatory T cells (Tregs), which are pivotal for suppressing excessive immune responses. Mechanistically, butyrate was found to enhance histone H3 acetylation at the promoter and conserved non-coding sequence regions of the Foxp3 gene locus. By upregulating Foxp3, a master regulator of Treg function, butyrate fosters immunological homeostasis.

Consequently, the depletion of SCFA-producing taxa observed in neurodegenerative patients may result in a concurrent impairment of protective mechanisms: a failure to resolve inflammation via GPR43 and a reduction in Treg-mediated immunosuppression due to impaired epigenetic regulation.

### 2.2. Secondary Bile Acids and the Gut–Liver–Brain Axis

Bile acids (BAs) are not merely digestive surfactants but potent signaling molecules regulating lipid and glucose metabolism, and immune responses. Primary BAs (e.g., cholic acid, CA), synthesized in the liver from cholesterol, undergo extensive biotransformation by the GM. Specific anaerobic bacteria, particularly *Clostridium scindens* and *Extibacter* species that express the *bai* (bile acid inducible) operon, perform 7 α-dehydroxylation, converting primary BAs into secondary BAs, such as deoxycholic acid (DCA) and lithocholic acid (LCA) [15,16,17].

Unlike their primary counterparts, secondary BAs are highly hydrophobic. This physicochemical property increases their propensity to influence the CNS, particularly under conditions of compromised BBB integrity [18]. Under physiological conditions, BAs act as ligands for nuclear receptors, including the farnesoid X receptor (FXR) and the membrane-bound G-protein-coupled bile acid receptor 1 (GPBAR1, also known as TGR5). These receptors function as critical hubs for metabolic and inflammatory signaling. Activation of TGR5 in microglia and monocytes typically suppresses the nuclear factor kappa-light-chain-enhancer of activated B cells (NF-κB) pathway, thereby reducing neuroinflammation [16].

However, this delicate balance is disrupted in neurodegenerative states. In AD, a profound shift in the bile acid profile has been documented. A large-scale metabolomic study involving 1464 subjects revealed significantly lower serum levels of primary CA and elevated levels of bacterially produced secondary DCA in AD patients compared to cognitively normal controls [15]. The ratio of DCA to CA (DCA:CA), which serves as a proxy for gut bacterial 7-α-dehydroxylase activity, was strongly associated with cognitive decline (P = 1.53 × 10^−8^) [15]. Furthermore, genetic variants in immune-related genes, such as *ABI3* and *MEF2C*, were linked to these altered BA profiles, suggesting a complex interplay between host genetics and microbiome function [15].

Excessive levels of cytotoxic secondary BAs, particularly DCA, may be detrimental to neuronal health. Due to their amphipathic nature, they have the potential to destabilize neuronal membranes and induce oxidative stress. Mechanistic studies indicate that hydrophobic BAs may compromise the BBB integrity via Rac1-dependent mechanisms [18] and disrupt mitochondrial membranes, leading to the release of reactive oxygen species (ROS) and the initiation of apoptosis [19]. This suggests a potential molecular link where gut-derived enzymatic activity may exacerbate neurodegeneration.

### 2.3. The Tryptophan-Kynurenine Pathway and Neurotoxicity

Under physiological conditions, the majority of dietary tryptophan (>95%) is metabolized via the kynurenine pathway (KP), primarily in the liver, leading to the production of nicotinamide adenine dinucleotide (NAD^+^), an essential cofactor for cellular energy metabolism [20,21]. Only a minor fraction is metabolized directly by gut bacteria into indole derivatives, such as indole and indole-3-propionic acid (IPA). However, under pathological conditions characterized by chronic stress or immune activation, this balance is disrupted [22].

Microbial indole derivatives exert neuroprotective effects via specific molecular targets. They serve as ligands for the aryl hydrocarbon receptor (AhR), a ligand-activated transcription factor involved in barrier function and immune regulation [23,24]. Crucially, indoles possess intrinsic redox properties. This chemical characteristic allows them to function as potent antioxidants, scavenging free radicals and modulating oxidative stress within the CNS [25]. Thus, the gut-derived indole pathway represents a direct mechanism by which the microbiota may bolster neuronal resistance to oxidative damage.

The rate-limiting step of the KP is catalyzed by the enzymes indoleamine 2,3-dioxygenase (IDO) and tryptophan 2,3-dioxygenase (TDO). Pro-inflammatory cytokines, particularly interferon-γ (IFN-γ), are potent activators of IDO activity, while stress hormones (glucocorticoids) activate TDO [20,22]. This enzymatic activation results in the rapid depletion of tryptophan and accumulation of kynurenine (KYN). Consequently, the availability of tryptophan for serotonin synthesis is reduced, which may contribute to mood disturbances often comorbid with neurodegenerative diseases [22].

**Figure 2:**
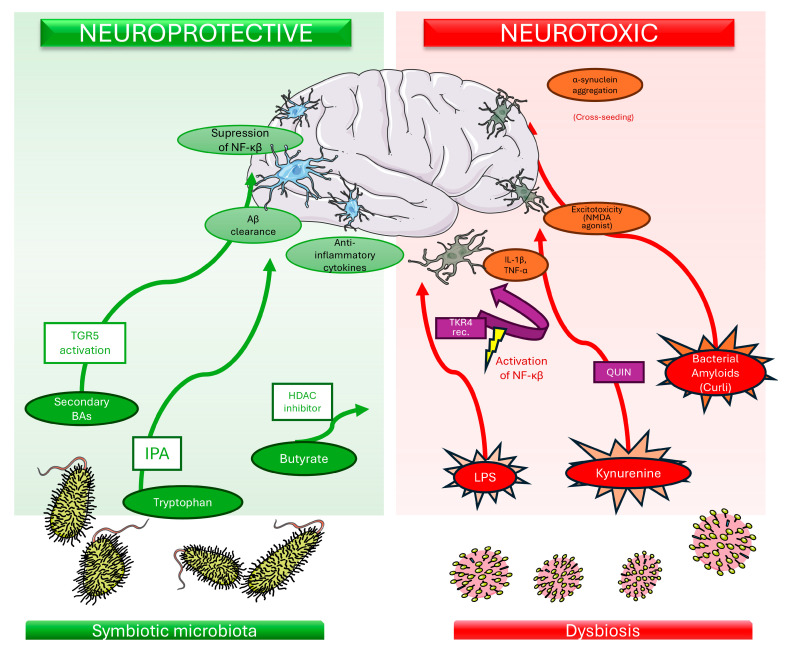
Proposed molecular mechanisms of microbiota-derived metabolites in neurodegeneration. Left panel (Neuroprotective): Commensal gut bacteria produce short-chain fatty acids (SCFAs, e.g., butyrate), which can inhibit histone deacetylases (HDAC) and modulate inflammatory responses. Indole derivatives derived from tryptophan metabolism and specific bile acid signaling via the TGR5 receptor are associated with maintenance of barrier integrity and neuroprotective pathways. Right panel (Neurotoxic): Dysbiosis is associated with increased release of lipopolysaccharides (LPS), which activate Toll-like receptor 4 (TLR4)/NF-κB signaling and promote the production of pro-inflammatory cytokines. A metabolic shift toward the kynurenine pathway favors the generation of neurotoxic metabolites such as quinolinic acid (QUIN). In addition, bacterial amyloids (e.g., Curli) may facilitate pathological protein aggregation through cross-seeding mechanisms. Images adapted from Servier Medical Art, licensed under CC BY 4.0.

The pathway further bifurcates into two distinct branches producing metabolites with opposing properties: the neuroprotective kynurenic acid (KYNA) and the neurotoxic 3-hydroxykynurenine (3-HK) and quinolinic acid (QUIN) [21] (see Figure 2). KYNA acts as an antagonist of N-methyl-D-aspartate (NMDA) receptors and α7 nicotinic acetylcholine receptors, offering protection against excitotoxicity [20]. Conversely, QUIN is a potent NMDA receptor agonist. Its accumulation represents a classic example of molecular neurotoxicity, leading to excitotoxic neuronal death, severe oxidative stress, and mitochondrial dysfunction [21,26].

In neurodegenerative diseases such as AD, PD, Huntington’s disease (HD), and ALS, a documented shift towards the neurotoxic branch of the pathway is observed [21,26]. A recent meta-analysis confirmed that blood levels of tryptophan are significantly lower in patients with AD, PD, and HD compared to healthy controls (HC), reflecting enhanced degradation via the KP [26]. Furthermore, an elevated KYN/tryptophan (K/T) ratio is frequently observed and serves as a robust biomarker of systemic inflammation and IDO activity [27]. In ALS, elevated levels of QUIN and 3-HK in the cerebrospinal fluid and blood serum correlate with neuroinflammation and excitotoxicity, while depletion of NAD^+^ due to the pathway dysregulation may contribute to metabolic failure in motor neurons [26].

Moreover, the GM play a crucial role in regulating this pathway. Dysbiosis can influence the host’s immune system, specifically inducing T helper 17 (Th17) cells, the resulting release of pro-inflammatory cytokines stimulates IDO, thereby perpetuating a cycle of inflammation and neurotoxicity [22].

### 2.4. Trimethylamine N-Oxide (TMAO)

Trimethylamine N-oxide (TMAO) represents a unique class of host-microbial co-metabolites produced through the diet-microbiota-liver axis. The process begins with the ingestion of dietary precursors containing a trimethylamine moiety, such as L-carnitine (abundant in red meat), choline, and phosphatidylcholine (found in eggs and dairy). As elucidated by Koeth et al. [28], GM metabolize these precursors into trimethylamine (TMA), which is subsequently absorbed into the portal circulation and oxidized in the liver by flavin-containing monooxygenases (FMO3) to form TMAO.

While elevated plasma TMAO is a well-established risk factor for atherosclerosis and cardiovascular disease [28], its implication in CNS pathology is more complex. The role of TMAO in neurodegeneration remains controversial and is primarily supported by associative data. Clinical studies have noted elevated TMAO levels in the cerebrospinal fluid and plasma of patients with AD [29] and PD [30], correlating with markers of synaptic degeneration and disease severity.

Despite the correlative nature of human studies, recent experimental models have proposed potential molecular mechanisms. Li et al. [31] demonstrated that TMAO may contribute to brain aging and cognitive impairment in SAMP8 mice. Mechanistically, they observed that TMAO treatment was associated with mitochondrial dysfunction and elevated production of reactive oxygen species (ROS) in the hippocampus. Furthermore, TMAO may potentially inhibit the mTOR signaling pathway, leading to the downregulation of synaptic plasticity-related proteins, including synaptophysin (SYN) and postsynaptic density protein 95 (PSD-95) [31].

These findings suggest that TMAO might not act merely as a bystander but could potentially exacerbate neuronal senescence under specific conditions. However, it remains to be determined whether TMAO is a causative agent driving neurodegeneration in humans or merely a biomarker of metabolic dysregulation and altered gut flora. Thus, further longitudinal studies are required to clarify its direct impact on neuronal health.

### 2.5. Lipopolysaccharides (LPS) and Microbial Amyloids: Inflammatory Signaling and Cross-Seeding

Lipopolysaccharides (LPS), integral components of the outer membrane of Gram-negative bacteria (e.g., *Escherichia coli*, *Bacteroides fragilis*), act as potent pro-inflammatory mediators. Under conditions of dysbiosis and compromised intestinal barrier integrity (“leaky gut”), LPS may translocate into the systemic circulation. Upon reaching the CNS, LPS is hypothesized to interact with microglia, the resident immune cells of the brain. The primary molecular mechanism involves the binding of LPS to the TLR4 complex, often requiring the co-receptor CD14 as depicted in Figure 2. This interaction triggers the nuclear factor kappa-light-chain-enhancer of activated B cells (NF-κB) signaling pathway, and the mitogen-activated protein kinase (MAPK) pathway [32,33].

The activation of these cascades results in the transcriptional upregulation and release of pro-inflammatory cytokines, such as IL-1β, IL-6 and TNF-α, which perpetuate neuroinflammation and may compromise neuronal survival [34]. Furthermore, LPS stimulation has been linked to the activation of the NLRP3 inflammasome in microglia, a process that further amplifies the inflammatory response via caspase-1 mediated cleavage of pro-IL-1β [35]. While elevated levels of LPS have been detected in the hippocampus of AD patients and substantia nigra of PD patients [36,37], it is crucial to note that evidence establishing a direct causal link between LPS and neurodegeneration is largely derived from animal and in vitro studies [34,38]. Consequently, the extent to which these mechanisms drive human neurodegenerative pathology, as opposed to being a secondary consequence of disease, remains under investigation.

However, a critical perspective on the role of LPS is necessary. While the “leaky gut” hypothesis is compelling, clinical measurements of circulating endotoxins remain technically challenging and prone to artifacts. Commonly used assays, such as the Limulus Amebocyte Lysate (LAL) test, are susceptible to interference from plasma lipids (e.g., triglycerides in metabolic disorders), β-glucans, and even contaminants in blood collection tubes, leading to false-positive results [39]. Furthermore, causality remains debated. As Brown and Heneka [40] emphasize, elevated systemic LPS is observed across diverse pathologies (e.g., sepsis, liver disease) and is insufficient to induce AD in isolation; moreover, rodents used in modeling are significantly less sensitive to endotoxin than humans, complicating translational interpretations. Thus, while LPS likely contributes to the inflammatory milieu, interpreting plasma endotoxin levels as primary driver of neurodegeneration requires caution and validation through more specific quantitative methods.

Beyond endotoxins, specific gut bacteria secrete functional amyloids, such as Curli fibers produced by *E. coli* and *Salmonella* spp. These bacterial proteins share distinct structural similarities with host amyloidogenic proteins, including β-sheets that are resistant to protease degradation. Through a mechanism known as “cross-seeding,” bacterial amyloids may potentially act as structural templates, accelerating the misfolding and aggregation of host proteins such as α-synuclein and amyloid-β [41,42]. Experimental models suggest that exposure to Curli-producing bacteria can enhance α-synuclein deposition in both the gut and the brain [32]. However, while the cross-seeding hypothesis offers a compelling molecular link between the microbiome and proteinopathies, current data are primarily experimental, and the physiological relevance of this mechanism in human disease progression requires further validation [43,44].

### 2.6. Microbiota-Derived Neurotransmitter-like Metabolites and Vagal Signaling

Although GABA and glutamate are classical neurotransmitters, increasing evidence indicates that gut microbiota–derived neurotransmitter-like metabolites can indirectly influence central nervous system function, primarily via vagal signaling [45]. The GM possesses the enzymatic machinery to synthesize canonical neurotransmitters, directly influencing the host’s neurochemistry. While early research focused on *Lactobacillus* and *Bifidobacterium* strains expressing glutamate decarboxylase (GAD) systems to convert glutamate into GABA, recent genomic and co-culture analyses have expanded this view. Strandwitz et al. [46] identified that specific genera, particularly *Bacteroides*, *Parabacteroides*, and *Eubacterium*, are prolific producers of GABA. Crucially, the relative abundance of fecal *Bacteroides* has been negatively correlated with brain signatures associated with depression (specifically, functional connectivity between the dorsolateral prefrontal cortex and the default mode network) in patients with major depressive disorder (MDD), suggesting that microbial GABA production is clinically relevant to human psychopathology [46].

However, a critical physiological challenge remains: Current evidence suggests that peripherally produced GABA may reach the CNS primarily under pathological conditions. Therefore, the central effects of gut-derived neurotransmitters are likely mediated through the stimulation of the vagus nerve (cranial nerve X), which serves as a major modulatory pathway for constitutive communication between the gut and the brainstem. This mechanism was elegantly elucidated by Bravo et al. [47], who demonstrated that chronic ingestion of *Lactobacillus rhamnosus* (JB-1) induced region-dependent alterations in GABA receptor expression in the mouse brain. Specifically, the probiotic treatment increased mRNA expression of the GABA (B1b) receptor in cortical regions while reducing it in the hippocampus, amygdala, and locus coeruleus, mirroring the effects of antidepressant pharmacotherapy.

Importantly, these neurochemical changes were accompanied by reduced stress-induced corticosterone levels and attenuated anxiety- and depression-related behaviors. The critical role of the neural pathway was confirmed when subdiaphragmatic vagotomy (severing the vagus nerve) completely abolished both the behavioral anxiolytic effects and the neurochemical receptor changes induced by the bacteria [47]. This confirms that the gut–brain axis relies heavily on vagal afferent signaling to translate microbial activity into central regulation of neurotransmission.

Dysbiosis-induced reduction in GABA-producing commensals, or an impairment in vagal signaling, may therefore disrupt the critical excitation/inhibition (E/I) balance in the CNS. A deficiency in inhibitory GABAergic signaling, coupled with potential glutamate accumulation driven by impaired microbial metabolism, lowers the threshold for excitotoxicity. This mechanism provides a tangible explanation for the motor deficits observed in PD and the cognitive agitation or anxiety frequently comorbid with AD [46,47].

A schematic overview of the microbiota-derived metabolites and molecular pathways discussed in this section is provided in Figure 2.

## 3. Literature Search Strategy and Selection Criteria

This article represents a narrative review; therefore, no formal PRISMA (preferred reporting items for systematic reviews and meta-analyses) guidelines were applied. A targeted literature search was performed using the PubMed and Scopus databases, covering publications available up to December 2025.

The search strategy employed a combination of keywords related to the gut microbiome, microbiota-derived metabolites, and neurodegenerative diseases, including “gut microbiota”, “gut–brain axis”, “microbiota-derived metabolites”, “short-chain fatty acids (SCFAs)”, “bile acids”, “tryptophan metabolism”, “lipopolysaccharides (LPS)”, “Alzheimer’s disease”, “Parkinson’s disease”, and “amyotrophic lateral sclerosis”.

Primary research articles employing metabolomics approaches (both targeted and untargeted) were prioritized to ensure the inclusion of direct experimental evidence. In addition, relevant review articles and meta-analyses were consulted to provide broader context and to identify key mechanistic pathways linking gut dysbiosis with central nervous system pathology.

## 4. Gut Dysbiosis in Alzheimer’s Disease

AD is characterized by a progressive cognitive decline, Aβ accumulation, and tau hyperphosphorylation. Emerging evidence suggests that gut dysbiosis may act as an important environmental contributor to AD pathology, influencing neuroinflammation through the MGB axis.

### 4.1. Mechanisms Linking Dysbiosis to AD Pathology

The condition of dysbiosis contributes to an increase in intestinal permeability, which enables pro-inflammatory agents to enter the bloodstream. This process can adversely affect the BBB and trigger neuroinflammation, a defining feature of AD [48]. The formation of tau proteins, another characteristic feature of AD, may be affected by GM, indicating a potential link between gastrointestinal health and neurodegenerative mechanisms [49].

SCFAs are produced through bacterial fermentation, primarily from carbohydrates reaching the distal gut, and serve as a crucial energy source for colonocytes [50]. However, metabolic analysis of fecal samples from AD patients reveals reduced mean concentrations of seven key SCFAs, including acetate and butyrate, compared to HC [51].

SCFAs, such as butyrate, generally support the integrity of the BBB. Mechanistically, butyrate acts as a potent histone deacetylase (HDAC) inhibitor. Sodium butyrate activates the phosphatidylinositol-3-kinase (PI3K)/protein kinase B (AKT)/cAMP-response element-binding protein (CREB)/brain-derived neurotrophic factor (BDNF) signaling pathway in microglia, leading to enhanced synaptic plasticity and the alleviation of MCI [52]. Furthermore, as butyrate may influence central nervous system function, potentially via indirect signaling pathways, it maintains activation of the vagus nerve and the hypothalamus [53,54]. Furthermore, butyrate has been shown to inhibit the aggregation of neurotoxic Aβ1–40 and Aβ1–42 peptides; in mouse models, sodium butyrate treatment reduced brain Aβ levels by as much as 40% [55].

Conversely, acetate, another SCFA, exhibits more complex and context-dependent. On one hand, studies have demonstrated that acetate supplementation reduces pro-inflammatory cytokine levels (such as TNF-α and IL-6) by down-regulating p38 mitogen-activated protein kinase (p38 MAPK) and NF-κB signaling, while simultaneously increasing anti-inflammatory cytokine concentrations (IL-4) through the up-regulation of transforming growth factor beta 1 (TGF-β1) signaling [56].

However, conflicting evidence exists regarding its net effect on neurodegeneration. While Huang et al. [50] point to a distinct neuroprotective mechanism via the reduction in neuroinflammation, other reports such as Bostick et al. [57] suggest the role of acetate is more ambiguous, noting that its elevated salivary concentrations can correlate with dementia status.

Mechanistically, acetate supplementation in the five familial AD (5xFAD) mice was associated with increased Aβ pathology, potentially by suppressing the microglial phagocytosis and clearance of Aβ [58]. Consistently, in humans, higher Aβ reactivity on positron emission tomography (PET) scans has been positively correlated with acetate levels [59].

TMAO has been associated with adverse cognitive outcomes Evidence suggests that TMAO exacerbates neuroinflammation and promotes astrocyte activation, thereby contributing to memory and learning deficits. Clinically, elevated TMAO levels correlate positively with established biomarkers of neurodegeneration, including phosphorylated tau protein, total tau (t-Tau), and neurofilament light chain (NFL) [60,61,62,63,64]. These pathological effects are potentially mediated by the induction of mitochondrial oxidative stress and subsequent synaptic damage.

Gut microorganisms are capable of regulating the production and synthesis of tryptophan derivatives. Certain derivatives, particularly IPA, may exert neuroprotective effects by acting as ligands for the aryl hydrocarbon receptor (AhR). IPA is capable of reaching the CNS, where it potentially prevents Aβ aggregation while improving neuronal survival. It also may attenuates neuroinflammation [65,66,67].

Conversely, another tryptophan derivative, such as indoxyl sulfate (IS), is a uremic toxin that represents a detrimental metabolite that may accumulate in the CNS under pathological conditions. IS induces oxidative stress, thereby triggering pro-inflammatory signaling in astrocytes [68]. Furthermore, a shift in tryptophan metabolism from the serotonin pathway toward the accumulation of quinolinic acid (QUIN), a potent NMDA receptor agonist, has been observed. This dysregulation is strongly associated with excitotoxicity, gray matter atrophy, and tau hyperphosphorylation [69].

LPS is a potent endotoxin primarily found in the outer membrane of Gram-negative bacteria, such as *Bacteroides fragilis*, *Escherichia coli*, and *Shigella flexneri* [70,71]. In the context of AD, LPS exerts a distinctly detrimental effect on neuronal health. Notably, LPS levels in the hippocampus of AD patients have been observed to be up to 26-fold higher than in HC, often accumulating in the perinuclear region of neurons [36].

Mechanistically, the pathogenic action of LPS is mediated by its interaction with the TLR4 complex. This binding triggers the NF-κB signaling pathway and potentially activates the NLRP3 inflammasome in microglia. This activation unleashes a pro-inflammatory cascade, releasing cytokines such as TNF-α and IL-1β. These pro-inflammatory mediators are hypothesized to compromise barrier integrity and perpetuate a chronic neuroinflammatory state, thereby potentially facilitating tau hyperphosphorylation and the accumulation of Aβ [72,73].

### 4.2. Alterations in Bacterial Composition: Clinical Evidence

A study of 25 AD and 25 HC individuals revealed that the GM of AD patients exhibits reduced microbial diversity and differs in composition from that of age- and sex-matched HC. Differences in bacterial counts were also identified. The microbiome of AD patients is characterized by a reduced population of *Firmicutes* bacteria, a reduced population of *Bifidobacterium* bacteria, and an increased population of *Bacteroidetes* bacteria [29]. These compositional shifts are consistent with a reduced capacity for SCFA production and a compromised intestinal barrier.

Another study suggests that, in AD patients, specific GM taxa may contribute to brain inflammation. The study measured the abundance of certain bacteria, including *Escherichia*/*Shigella* and *Pseudomonas aeruginosa*, which are associated with pro-inflammatory responses. The study hypothesized that specific GM taxa might induce immune responses that lead to Aβ deposition, a hallmark of AD. This indicates a complex interaction between gut health and brain pathology. Among the participants, specific analyses were conducted on the GM of 40 cognitively impaired amyloid-positive patients, 33 cognitively impaired amyloid-negative patients, and 10 HCs. The study highlights that patients with cognitive impairment and brain amyloidosis have a lower abundance of the anti-inflammatory bacterium *Eubacterium rectale* and a higher abundance of the pro-inflammatory bacterium *Escherichia*/*Shigella* compared to HC and the patients without amyloidosis [74]. This suggests that the overgrowth of pro-inflammatory taxa may directly contribute to peripheral inflammation and subsequent amyloid deposition.

A subsequent study, conducted on a group of 43 AD patients and 43 age- and gender-matched HC, showed that the GM composition in AD patients is notably different from that of HC. Specific bacterial taxa, including *Bacteroides*, *Actinobacteria*, *Ruminococcus*, *Lachnospiraceae*, and *Selenomonadales*, were identified as having different abundances between the two groups. The diversity of GM was found to be reduced in AD patients. *Bacteroides fragilis*, which has anti-inflammatory properties, was found to be less abundant in AD patients. Its low abundance may compromise the intestinal barrier, potentially facilitating the translocation of pro-inflammatory bacterial endotoxins, particularly lipopolysaccharides (LPS), into the systemic circulation. In contrast, *Ruminococcus* species were found to be increased in patients with AD. These bacteria require fermentable carbohydrates and can degrade mucus, which may have implications for gut health and inflammation [75]. Collectively, these taxonomic alterations point towards a functional shift from a homeostatic to a pro-inflammatory metabolic output.

Moving beyond taxonomy to functional metagenomics, Haran et al. [76] provided critical mechanistic insight by linking dysbiosis to intestinal epithelial homeostasis. They demonstrated that the microbiome of AD patients, characterized by a depletion of butyrate-producing *Eubacterium* and *Roseburia*, induced significantly lower expression of P-glycoprotein (P-gp) in intestinal epithelial cells compared to non-demented controls. Since P-gp is a key mediator of intestinal homeostasis and toxin efflux, its downregulation suggests a direct pathway by which the AD microbiome compromises the intestinal barrier and promotes systemic inflammation.

Crucially, recent studies have moved beyond simple abundance profiling to link specific microbial shifts with clinical disease severity. For instance, Vogt et al. [29] demonstrated that the abundance of Bacterioides in AD patients was positively correlated with cerebrospinal fluid (CSF) levels of YKL-40, a marker of neuroinflammation, and negatively correlated with the Aβ42/Aβ40 ratio. Furthermore, MahmoudianDehkordi et al. [16] reported that an increased ratio of secondary to primary bile acids (DCA:CA)—a metabolic signature of gut dysbiosis—was strongly associated with worse cognitive performance (ADAS-Cog13 scores) and brain atrophy, suggesting that gut-mediated metabolic dysregulation tracks with disease progression. Nevertheless, since these correlations were observed in patients with established dementia, it remains to be determined whether these metabolic shifts are primary drivers of neurodegeneration or secondary consequences of the dietary and lifestyle changes inherent to cognitive decline.

### 4.3. Critical Analysis of AD Microbiome Studies

Synthesizing the available data, a reduction in microbial diversity and a depletion of anti-inflammatory taxa, particularly *Faecalibacterium* and *Bifidobacterium*, appear to be the most consistent signatures of AD across different cohorts. These alterations strongly correlate with the clinical severity of cognitive decline. However, discrepancies exist regarding specific genera; for instance, *Bacteroides* abundance was reported as increased by Vogt et al. [29] but decreased by Zhuang et al. [75]. Such contradictions likely stem from differences in geographic location and dietary habits, which were not uniformly controlled in these studies. Furthermore, as most studies focused on patients with established dementia, it remains unclear whether these dysbiotic patterns are a cause or a consequence of the altered lifestyle associated with AD. Consequently, metabolite profiling may provide more functionally relevant biomarkers than microbiota composition alone.

In summary, while human cohorts consistently exhibit a depletion of anti-inflammatory taxa, the heterogeneity in reported pathobionts across studies highlights the influence of environmental confounders. This contrasts with controlled animal models, in which specific bacterial inoculations drive clear amyloid and tau pathology [77,78], suggesting that human data currently reflect a complex interaction between disease and lifestyle rather than a simple causal chain.

Current findings regarding AD microbiota alterations are summarized in Table 1.

## 5. Gut Dysbiosis in Parkinson’s Disease

Parkinson’s disease (PD) is a progressive neurodegenerative disorder characterized by motor symptoms, including bradykinesia, rigidity, and resting tremor, as well as a wide spectrum of non-motor manifestations. Growing evidence indicates that gut dysbiosis may contribute to PD pathophysiology by modulating neuroinflammatory processes, α-synuclein aggregation, and gut–brain communication via the microbiota–gut–brain axis [79].

### 5.1. Pathophysiological Implications of Dysbiosis in PD

The GM and its metabolites have been increasingly implicated in the pathophysiology of PD. The first reports in this area were published in 2015 by Scheperjans et al. [37], who observed a marked decrease in *Prevotellaceae* abundance in the stool of PD patients compared to HC. A positive link was also observed between *Enterobacteriaceae* abundance and the severity of postural instability and gait difficulty [37]. In subsequent years, another study found that PD patients exhibit reduced levels of SCFAs, which are key mediators for maintaining a proper gut barrier [80,81].

Emerging evidence suggests that the microbiota may influence the synthesis and aggregation of α-synuclein. Bacteria such as *Escherichia coli* release extracellular amyloid proteins, including Curli fibers. In animal studies, these bacterial amyloids have been shown to induce α-synuclein deposition in both the gut and the brain via cross-seeding mechanisms [32,41,42,43,44]. However, it is crucial to note that most evidence regarding bacterial amyloids is derived from experimental models, and their relevance to human PD pathology remains under investigation.

Gastrointestinal dysfunction is a prodromal feature commonly observed in individuals with PD, with approximately 60 to 80% reporting constipation [82]. Constipation is associated with the accumulation of α-synuclein and neurodegenerative processes in the ENS [83]. This observation supports the hypothesis of a “gut-first phenotype,” suggesting that pathology may originate in the ENS in a subset of patients before spreading to the CNS via the vagus nerve. In addition, increased local inflammation and oxidative stress, along with associated increased intestinal permeability, are present in PD [84].

Additionally, the role of LPS is critical. Dysbiosis in PD often leads to increased intestinal permeability, commonly referred to as “leaky gut”, facilitates the translocation of LPS and other pro-inflammatory metabolites into the systemic circulation, potentially triggering immune responses and initiating inflammation that may precede neurological symptoms [34,38,85,86,87]. Experimental data suggest that LPS may promote α-synuclein aggregation, as suggested by experimental models involving Gram-negative bacteria.

Moreover, alterations in the GM compromise the production of neuroprotective SCFAs. When the abundance of SCFA-producing bacteria is reduced, as seen in PD, the loss of these protective metabolites contributes to the progression of neurodegeneration [80]. Cytokines also play a crucial role in PD pathogenesis. In colon biopsies of PD patients, gene upregulation for cytokines such as IFN-γ, IL-1β, IL-6, IL-8, and IL-17A can be observed. Furthermore, chronic low-grade inflammation was detected in the form of elevated TNF, IL-6, IL-1β, and the chemokine C-C motif chemokine ligand 5 (CCL5) in blood serum and cerebrospinal fluid even before the development of neurological symptoms, which may suggest a role for these cytokines in disease escalation [33,88,89].

### 5.2. Microbial Signatures and Clinical Correlations

Interestingly, the abundance of *Prevotellaceae* was found to be reduced by almost 80% among PD patients compared to HCs [37]. It was also shown that the relative abundance of *Enterobacteriaceae* correlated positively with the severity of postural instability and gait problems. The study included 72 people with PD and 72 HC sex- and age-matched. These findings suggest an altered gut microbiome in PD [37]. Since *Prevotella* species are key mucin degraders and SCFA producers, their depletion, combined with the expansion of pro-inflammatory *Enterobacteriaceae*, supports a mechanism of compromised barrier function and systemic endotoxemia.

Beyond diagnostic differentiation, specific gut taxa have been associated with motor symptom severity and phenotypic progression. Scheperjans et al. [37] reported that the relative abundance of *Enterobacteriaceae* was positively correlated with the severity of postural instability and gait difficulty (PIGD), a phenotype often associated with faster disease progression. This suggests that specific microbial shifts might track with disease evolution, potentially influencing the clinical trajectory from tremor-dominant to more debilitating gait-impairment stages, although the cross-sectional nature of the study precludes determining whether this bacterial enrichment precedes motor decline or merely results from altered intestinal motility associated with the PIGD phenotype.

Further validation of these inflammatory signatures was provided by Keshavarzian et al. [90], who observed a marked reduction in anti-inflammatory, butyrate-producing genera (*Blautia*, *Coprococcus*, *Roseburia*) in PD feces, alongside an enrichment of pro-inflammatory *Ralstonia* in the mucosa. Importantly, recent studies have addressed the potential cofounders, such as medication and shared environments. Bedarf et al. [91] confirmed that dysbiosis (e.g., decreased *Prevotella* and *Eubacterium*) persists in early-stage, L-DOPA-naïve patients, suggesting these alterations are disease-intrinsic rather than drug-induced. Similarly, Zhang et al. [92] compared PD patients with their healthy spouses to control for dietary and household factors. A distinct microbial signature was identified in patients (including increased *Akkermansia* and *Bilophila*) that accurately differentiated them from both spouses and unrelated controls, reinforcing the link between specific gut taxa and PD pathology

A subsequent study was conducted on 197 PD individuals and 130 HC to determine whether PD is associated with gut microbiome dysbiosis [93]. Significant changes in abundance of the families *Bifidobacteriaceae*, *Christensenellaceae*, *Tissierellaceae*, *Lachnospiraceae*, *Lactobacillaceae*, *Pasteurellaceae*, and *Verrucomicrobiaceae* was found. Furthermore, changes in many pathways were identified, including those related to the metabolism of plant compounds and xenobiotic degradation [93]. The depletion of these taxa, particularly *Lachnospiraceae*, points towards a diminished capacity for butyrate synthesis, which is essential for suppressing neuroinflammation.

Further analysis suggests that specific operational taxonomic units (OTUs) were found to be significantly more abundant in patients with PD compared to HCs [94]. The study involved a total of 175 participants, including 76 patients with PD, 26 patients with idiopathic rapid eye movement sleep behavior disorder (RBD), and 78 HCs. Notably, OTUs from the genera *Akkermansia* and *Prevotella* were more abundant in PD patients with RBD compared to those without RBD, suggesting a potential link between these taxa and PD symptoms. Moreover, the GM of PD patients showed significant relationships with nonmotor symptoms, as assessed by the movement disorder society-unified Parkinson’s disease rating scale (MDS-UPDRS). Specifically, OTUs from *Anaerotruncus*, *Clostridium* XIVa, and *Lachnospiraceae* were associated with motor symptoms [94]. These associations indicate that specific microbial signatures may track with distinct clinical sub-phenotypes, such as RBD, rather than disease presence alone.

One of the critical questions is what causes microbiome dysbiosis in PD. One hypothesis is based on the increased activity of pathways that degrade xenobiotics, such as atrazine and chloroalkanes. This hypothesis states that exposure to pesticides and herbicides in the agricultural environment or drinking water from a well may increase the risk of developing PD [95]. This suggests that environmental toxins may drive dysbiosis, creating a feedback loop that impairs the microbiota’s ability to detoxify xenobiotics, thereby increasing neuronal exposure to these compounds.

### 5.3. Critical Analysis of PD Microbiome Studies

In the context of PD, the reduction in *Prevotellaceae* and members of the *Lachnospiraceae* family stands out as a highly reproducible finding across studies [37,93], linking gut dysbiosis directly to reduced mucin synthesis and increased intestinal permeability. The consistent enrichment of *Akkermansia* reported in later studies [93,94] presents a paradox, as this bacterium is typically considered beneficial; yet, in PD, it may reflect a compensatory response or altered mucin layer dynamics.

A major methodological limitation in these studies is the potential confounding effect of PD medications, particularly bacterial metabolism of levodopa, which was not fully adjusted for in earlier analyses. Furthermore, variations in dietary habits and gastrointestinal transit time (constipation) represent additional confounding variables that may obscure the true microbial signature of the disease. Consequently, distinguishing between microbiome changes driven by disease pathology versus those induced by pharmacotherapy or lifestyle remains a critical challenge. Collectively, PD microbiota alterations correlate strongly with motor symptom severity. However, distinguishing whether these shifts are primary drivers of neurodegeneration or secondary consequences of gastrointestinal autonomic dysfunction requires further longitudinal validation, distinct from the direct causation observed in animal models [96].

Current findings regarding alterations in PD microbiota are summarized in Table 2.

## 6. Gut Dysbiosis in Amyotrophic Lateral Sclerosis

ALS is a progressive and fatal neurodegenerative disorder characterized by the degeneration of upper and lower motor neurons, leading to muscle weakness, paralysis, and respiratory failure. Increasing evidence suggests that alterations in GM composition and function may contribute to ALS pathophysiology by modulating systemic inflammation, metabolic homeostasis, and immune responses along the gut–brain axis [97].

### 6.1. Metabolic and Immune Dysregulation in ALS

In ALS, the gut–brain axis has been implicated in modulating disease progression. Available data remain more heterogeneous compared to AD or PD. In some cohorts, alterations in the gut bacterial population have been reported, characterized by a potential decrease in anti-inflammatory genera such as *Faecalibacterium* and *Lactobacillus*, alongside an enrichment of pro-inflammatory taxa, such as *Proteobacteria* and *Escherichia coli*. This imbalance, known as dysbiosis, is hypothesized to drive systemic inflammation, contributing to the development of neuroinflammation [29].

One of the mechanisms by which gut inflammation may exacerbate neurodegenerative processes is increased oxidative stress, which disrupts mitochondrial function. Additionally, a reduction in the abundance of bacteria capable of producing SCFAs has been noted in several studies [98]. Dysbiosis may therefore contribute to the altered neurotransmission observed in ALS, potentially affecting motor neuron function and survival [99].

Impaired intestinal barrier integrity, or “leaky gut”, is driven by the reduction in tight junction proteins, such as zonula occludens-1 (ZO-1), and E-cadherin [100]. Such disruption facilitates endotoxemia—the translocation of LPS into the bloodstream. Crucially, elevated systemic LPS levels have been associated with a more rapid disease progression and a greater burden of systemic immune activation [101].

Furthermore, ALS is characterized by increased oxidative stress, significant mitochondrial damage, and disturbed glucose metabolism. Motor neurons are particularly vulnerable to energy deficits; consequently, dysbiosis-associated metabolic disturbances have been linked to decreased levels of nicotinamide, a precursor of NAD^+^ essential for energy metabolism, which has been correlated with poorer motor outcomes [102]. The reduction in butyrate is also crucial for the immune dysregulation observed in ALS, specifically the imbalance between regulatory T cells (Tregs) and Th17 cells. This loss is associated with the exacerbation of neuroinflammation and elevated levels of pro-inflammatory cytokines, such as IL-17 and IL-23 [103].

### 6.2. Diversity and Compositional Shifts

A study involving 50 ALS patients and 50 age- and sex-matched HC revealed specific variations in intestinal microbial composition. It has been reported that ALS patients exhibited a higher abundance of *Escherichia coli* and *Enterobacteria*, while total yeast was less abundant [104]. Although differences in microbial profiles were noted, the study suggested that a clear dysbiosis status, defined as a global disruption of community structure, was not evident in ALS patients. That indicates that microbiota alterations in ALS may be characterized by specific taxonomic shifts (e.g., pathobiont enrichment) rather than a widespread collapse of the ecosystem.

Consistent with findings in the cohorts, Zeng et al. [105] reported a significant increase in *Bacteroidetes* and a reduction in *Firmicutes* (specifically *Meganomas*) in ALS patients. Crucially, their combined metagenomic and metabolomic analysis revealed that these taxonomic shifts were accompanied by a downregulation of metabolic pathways involved in amino acid and carbohydrate metabolism, suggesting a functional impairment of the microbiome that extends beyond simple abundance changes.

However, findings across cohorts remain inconsistent, likely due to differences in disease severity and methodology. For instance, Brenner et al. [106] compared 25 ALS patients with high functional status and strict inclusion criteria with 32 age- and gender-matched controls. In contrast to smaller pilot studies, they observed no substantial alteration in the overall composition, diversity, or predicted metagenomes of the GM, with the exception of a minor difference in uncultured *Ruminococcaceae*. This suggests that gut dysbiosis might not be a universal feature in the early stages of ALS but could develop progressively. Consequently, this discrepancy highlights the critical importance of sample size and the rigorous control of confounding variables, such as BMI loss and dietary changes, to distinguish primary disease mechanisms from secondary effects.

More recently, a comprehensive study involving 75 ALS patients (43 male, 32 female) and 110 healthy control participants (44 male, 66 female) demonstrated significantly reduced α-diversity (*p* < 0.01) and distinct phylum-level shifts, with notable reductions in *Firmicutes* and elevations in *Cyanobacteria* compared to controls [107]. Genus-level analysis highlighted a depletion of beneficial butyrate-producing taxa such as *Faecalibacterium* and *Bifidobacterium* concurrent with an increase in *Bacteroides* and *Parasutterella*. Crucially, integrative analysis revealed strong correlations between these specific bacterial taxa and altered plasma lipid metabolites, suggesting a mechanistic link between gut dysbiosis and systemic metabolic dysregulation in ALS [107].

In the context of disease progression, Guo et al. [107] recently provided compelling evidence linking the gut microbiome to functional decline. Their longitudinal analysis revealed that specific microbial modules—characterized by depletion of *Akkermansia* and specific *Lachnospiraceae* taxa—were significantly correlated with lower ALS Functional Rating Scale-Revised (ALSFRS-R) scores. Moreover, the serum metabolomic profile associated with these microbial shifts (specifically altered lipid metabolism) tracked with the rate of functional decline over time, supporting the hypothesis that the microbiome-metabolome axis may modify the speed of neurodegeneration in ALS. However, it must be noted that these findings are derived from diagnosed patients where neurodegenerative changes are already established. Consequently, it remains difficult to definitively resolve whether this dysbiosis is a driver of functional decline or a secondary metabolic adaptation to the hypermetabolic state inherent to ALS pathology.

Compelling evidence from animal models further underscores the functional impact of the microbiome. In ALS-prone Sod1 transgenic (Sod1-Tg) mice, significant dysbiosis was observed, which notably depended on the vivarium environment [102]. This composition shift was mechanistically linked to altered systemic metabolite configurations, particularly nicotinamide availability. Significantly, the study demonstrated that distinct commensal bacteria could modulate disease progression in these animals. While supplementation with *Akkermansia muciniphila* (AM) ameliorated symptoms, colonization with *Ruminococcus torques* and *Parabacteroides distasonis* exacerbated pathology. These findings suggest that specific gut taxa may exert distinct protective or deleterious effects on ALS progression via metabolic modulation [102].

High-resolution metagenomic sequencing provided further mechanistic insights into ALS dysbiosis. Nicholson et al. [98] revealed that while overall microbial diversity remained stable, there was a profound functional depletion of key butyrate-producing species, specifically *Roseburia intestinalis* and *Eubacterium rectale*. This reduction remained significant even after adjusting for confounding factors such as constipation. Mechanistically, the loss of these taxa implies a diminished capacity for colonic butyrate synthesis, potentially compromising histone deacetylase (HDAC) inhibition and regulatory T-cell (Treg) induction, both of which are neuroprotective pathways impaired in ALS.

Conversely, the study identified an enrichment of pro-inflammatory taxa, including *Escherichia* and *Streptococcus salivarius*. This distinct compositional profile suggests a compounded pathogenic mechanism, characterized by the simultaneously depletion of neuroprotective metabolites (butyrate) and the expansion of potential sources of systemic endotoxemia. Statistical modeling confirmed that the total abundance of butyrate producers was strongly associated with a lower risk of ALS, highlighting the potential of the gut microbiome as a modifier of disease susceptibility [98].

Synthesizing these findings, it is crucial to acknowledge that a uniform taxonomic ALS signature has not yet been established, likely due to the substantial heterogeneity observed across cohorts. However, despite this taxonomic variability, a functional convergence is evident. The data consistently point towards a metabolic imbalance: the loss of biosynthetic capacity for neuroprotective metabolites (e.g., butyrate, nicotinamide) coupled with an increased antigenic load from pro-inflammatory pathobionts. Such alterations contribute to systemic inflammation via reduced SCFA production. Collectively, current evidence supports a potential mechanistic link between GM composition and ALS pathophysiology, warranting further longitudinal and diet-controlled research [98]. Thus, ALS dysbiosis may be best defined by its functional consequences rather than a specific bacterial census.

### 6.3. Heterogeneity of Findings in ALS

While multiple studies confirm gut dysbiosis in ALS, the specific microbial alterations reported are highly heterogeneous, indicating the lack of a single, consistent “ALS microbiome signature”. For instance, while some studies report an enrichment of *Akkermansia muciniphila* potentially as a compensatory response [102], others do not observe this shift or focus on different taxa such as *Escherichia coli* [104]. This discrepancy may be attributed to significant methodological differences, including varying sample sizes (ranging from 20 to 185 participants), different sequencing techniques (16S rRNA vs. metagenomics), and the use of diverse control groups (healthy spouses vs. unrelated controls).

Crucially, environmental and clinical confounding factors represent a major challenge in interpreting these datasets. ALS patients frequently undergo significant dietary modifications (due to dysphagia), experience rapid BMI loss (hypermetabolism), and utilize various supplements, all of which act as potent modulators of the microbiome. Most existing studies have not fully adjusted for these variables. Furthermore, the clinical heterogeneity of ALS itself, encompassing variable progression rates, distinct sites of onset (bulbar vs. spinal), and diverse genetic backgrounds, likely contributes to the variability in microbiome profiles.

Finally, the majority of human studies are cross-sectional, which limits the ability to infer causality. Consequently, it remains difficult to distinguish whether dysbiosis is a driver of pathology or a secondary consequence of the lifestyle and physiological changes associated with disease progression. Current evidence in ALS points to a functional depletion of butyrate producers and to alterations in nicotinamide metabolism. However, unlike murine models where specific taxa directly modulate survival [102,108] human data remain associative and likely influenced by the disease-related metabolic hypermetabolism and dietary modifications inherent to bulbar dysfunction.

Current findings regarding alterations in ALS microbiota are summarized in Table 3.

**Figure 3:**
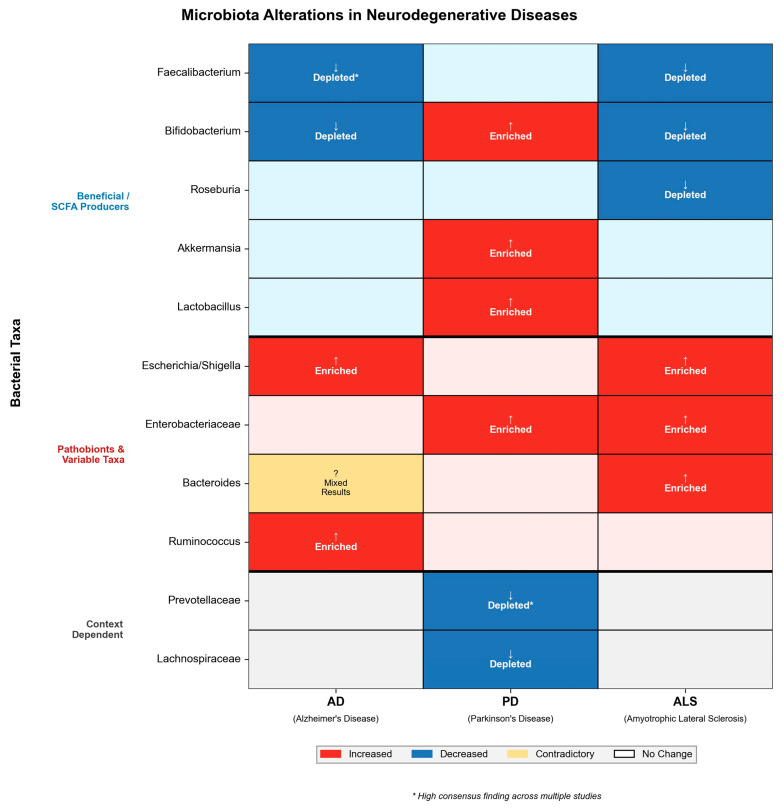
Heatmap summary of alterations in selected key bacterial taxa across Alzheimer’s disease (AD), Parkinson’s disease (PD), and amyotrophic lateral sclerosis (ALS). Red indicates enrichment, blue indicates depletion, while yellow denotes inconsistent findings across studies. The asterisk (*) marks taxa with high consensus across multiple independent cohorts. Bacterial taxa are functionally categorized into beneficial short-chain fatty acid (SCFA) producers, potential pathobionts, and context-dependent groups. Data were synthesized from studies reviewed in Section 4, Section 5 and Section 6.

A comparative overview of key bacterial taxa alterations across AD, PD, and ALS is summarized in Figure 3.

## 7. Therapeutic Implications and Challenges

### 7.1. Mechanisms-Driven Interventions

The transition from descriptive microbiome associations to precision therapeutics requires dissecting the specific molecular pathways linking gut dysbiosis to neurodegeneration. Current evidence suggests that targeting metabolic byproducts rather than taxonomic composition per se offers superior translational potential.

A breakthrough in mechanistic understanding was reported by Wang et al. [109], who demonstrated that gut dysbiosis in AD models promotes peripheral accumulation of phenylalanine and isoleucine. These amino acids act as metabolic cues that drive the differentiation and proliferation of pro-inflammatory Th1 cells. Crucially, the carbohydrate-based agent sodium oligomannate (GV-971) therapeutically remodeled the GM, suppressed these amino acid signatures and blocked Th1 infiltration into the brain, thereby inhibiting M1 microglial activation. This establishes a direct gut-metabolite-immune axis as a druggable target for ameliorating cognitive impairment.

Another distinct therapeutic mechanism involves mimicking microbiota-derived metabolites to protect motor neurons. In ALS, the therapeutic efficacy of taurusodiol combined with sodium phenylbutyrate underscores the approach. This combination mitigates endoplasmic reticulum (ER) stress and mitochondrial dysfunction, effectively slowing functional decline [110]. This suggests that metabolic postbiotics may bypass the need for viable bacterial colonization, offering a more pharmacokinetically stable intervention than live biotherapeutics.

Beyond single-molecule drugs, dietary modulation provides a broader metabolic impact. High adherence to the MIND diet is associated with reduced postmortem β-amyloid load [111]. Mechanistically, this likely relies on the high dietary fiber content (prebiotics), which is fermented by commensal bacteria into SCFAs such as butyrate. Butyrate functions as HDAC inhibitor and tightens the blood–brain barrier via upregulation of claudin-5, thereby reducing the influx of peripheral neurotoxins.

### 7.2. Microbiota-Derived Metabolites as Early Diagnostic Biomarkers

A critical question in neurodegeneration is whether microbial shifts precede clinical symptoms. Recent metabolomic profiling suggests that microbiota-derived metabolites may serve as prodromal biomarkers.

While the mechanism of amino acid-driven inflammation is clear, Wang et al. [109] further demonstrated its diagnostic utility. They identified that elevated plasma phenylalanine and isoleucine levels could distinguish patients with mild cognitive impairment (MCI) due to AD from healthy controls with high accuracy (AUC > 0.80). This suggests that specific metabolic signatures may serve as accessible blood-based biomarkers for neuroinflammatory risk prior to the onset of frank dementia.

In PD, gut alterations often precede motor symptoms by years. Specific markers include reduced fecal SCFA concentrations and an enrichment of opportunistic pathogens, such as *Desulfovibrio bacteria* (producers of toxic hydrogen sulfide) and *Enterobacteriaceae* [112]. These shifts correlate strongly with prodromal non-motor features, such as constipation and REM sleep behavior disorder (RBD), offering a potential window for early screening [113].

### 7.3. Critical Analysis of Limitations

Despite the promise of microbiome modulation, translational failure in clinical trials often stems fro a lack of ecological understanding—specifically, the failure to account for competitive dynamics and niche availability within the host gut.

Zmora et al. [114] fundamentally challenged the utility of empiric probiotics (a multi-strain preparation of *Lactobacillus*, *Bifidobacterium*, *Lactococcus*, and *Streptococcus*) by demonstrating that humans exhibit person-specific mucosal colonization resistance. Using invasive endoscopic sampling, they showed that probiotic shedding in stool often represents a transient “washout” rather than successful mucosal engraftment. Thus, without pre-treatment screening of the host microbiome to determine permissiveness, probiotic efficacy is unpredictable.

The common practice of administering probiotics to prevent antibiotic-associated dysbiosis may be counterproductive. Suez et al. [115] revealed that administering the same multi-strain preparation (*Lactobacillus*, *Bifidobacterium* spp.) after antibiotics significantly delayed the reconstitution of the indigenous microbiome and host transcriptome compared to spontaneous recovery. In contrast, autologous FMT induced a near-complete recovery within days. This highlights the superiority of ecologically compatible consortia (the host’s own adapted flora) over generic strains that may act as metabolic disruptors in a recovering ecosystem.

In FMT trials, clinical remission is often driven by specific “super-donors”. Wilson et al. [116] characterized these donors as having not only high diversity, but a specific enrichment of keystone species from the *Lachnospiraceae* and *Ruminococcaceae* families (major butyrate producers). This necessitates a shift from random donor selection to metabolically matched donor–recipient pairing to ensure the transfer of functionally critical taxa.

Future directions must move toward precision microbiome medicine, where patients are stratified based on their baseline enterotypes and metabolic profiles (e.g., responders vs. non-responders) before intervention. Current metabolite-targeted intervention strategies, their mechanistic targets, and clinical outcomes are summarized in Table 4.

## 8. Conclusions and Future Perspectives

### 8.1. Synthesis of Findings

In summary, emerging evidence suggests that gut dysbiosis appears to play a significant role in the pathogenesis and progression of neurodegenerative diseases such as AD, PD, and ALS. The complex interplay between the gut microbiome and the central nervous system, often referred to as the “microbiota–gut–brain axis” [1,5,6], appears to influence neuroinflammation, amyloid deposition, and neurodegeneration through various mechanisms. These include immune modulation, the production of neuroactive metabolites such as SCFAs [8,9], and impairment of blood–brain barrier (BBB) integrity mediated by factors like LPS [34,36].

Crucially, this review highlights that dysbiosis shifts metabolic outputs towards neurotoxicity. Rather than a single taxonomic signature, the unifying feature across these disorders is a functional imbalance: secondary bile acids (BAs) may compromise BBB integrity [18], tryptophan metabolism is diverted towards the neurotoxic kynurenine pathway [21,26], and reduced microbial GABA production disrupts the inhibitory/excitatory balance in the CNS [46].

In AD, the predominance of pro-inflammatory bacteria, such as *Escherichia*/*Shigella*, and the reduction in anti-inflammatory and butyrate-producing taxa, such as *Faecalibacterium* and *Bifidobacterium*, are consistently reported [29,75]. These shifts correlate with peripheral inflammation and amyloid pathology, potentially driving microglial activation and Aβ deposition via systemic endotoxemia [36,121].

In PD, a distinct pattern of gut dysbiosis is observed, characterized by the depletion of *Prevotella* and *Lachnospiraceae*, and the enrichment of *Enterobacteriaceae* and *Akkermansia*. These changes correlate with motor symptom severity and postural instability [37,98]. Mechanistically, this dysbiosis may increase intestinal permeability and promote α-synuclein aggregation through cross-seeding with bacterial amyloids such as Curli [32,86].

In ALS, alterations include the loss of beneficial taxa such as *Faecalibacterium*, *Roseburia*, and *Eubacterium rectale*, alongside enrichment of *Escherichia coli* and *Parasutterella* [98,104,107]. Such changes are mechanistically linked to disturbances in systemic lipid metabolism [107] and the depletion of neuroprotective metabolites such as nicotinamide, thereby potentially accelerating motor neuron degeneration [102].

### 8.2. Limitations and Methodological Challenges in Current Research

A fundamental limitation in the current literature is the predominance of cross-sectional designs, which hinders the determination of causality. Most studies recruit patients who are already diagnosed and symptomatic (“late-stage” analysis), making it difficult to discern whether dysbiosis is a primary driver of pathology or a secondary consequence of disease progression. Factors such as reduced mobility and gastrointestinal autonomic dysfunction significantly increase transit time, thereby altering microbial composition. Similarly, dietary modifications—such as the switch to soft foods due to dysphagia in ALS or altered appetite in AD—can profoundly reshape the microbiome. Crucially, chronic medication use represents another major confounder: for instance, levodopa in PD and anticholinergics in AD have been shown to directly alter microbial composition independent of the neurodegenerative process itself. Consequently, current data may capture a “disease-modified” microbiome rather than a “disease-causing” one. To resolve this, future research must prioritize prospective longitudinal studies of cognitively normal elderly populations to identify microbial shifts that authentically precede clinical diagnosis.

Furthermore, there is notable heterogeneity in study methodologies. Variations in sample collection, DNA extraction protocols, and sequencing platforms (e.g., 16S rRNA gene sequencing vs. shotgun metagenomics) render direct comparisons between cohorts challenging. Many studies involved relatively small sample sizes, particularly in the case of ALS, which likely contributes to inconsistent findings regarding specific bacterial taxa. Moreover, potential confounding factors—such as diet, long-term medication use (e.g., levodopa in PD), and comorbidities—were not uniformly controlled, potentially masking disease-specific alterations.

### 8.3. Directions for Future Investigation

To bridge the gap between correlation and causation, the field must undergo a paradigm shift from descriptive taxonomy to functional metabolomics. Future research should prioritize large-scale, prospective longitudinal cohort studies recruiting phenotypically normal elderly populations to determine whether specific microbial shifts precede the onset of neurological symptoms. Integrating high-resolution shotgun metagenomics with advanced metabolomics within these cohorts will be essential to identify functional signatures rather than just taxonomic lists. Specifically, gas chromatography-mass spectrometry (GC-MS) is recommended for the precise quantification of volatile SCFAs [122], while ultra-high performance liquid chromatography-tandem mass spectrometry (UHPLC-MS/MS) offers superior sensitivity for profiling non-volatile metabolites, such as secondary bile acids [16,107] and tryptophan derivatives [21]. Furthermore, combining these multi-omics approaches with machine learning algorithms could facilitate the identification of novel biomarkers for early diagnosis and disease monitoring [107].

Concurrently with clinical observations, molecular research must focus on elucidating the precise pathways by which bacterial metabolites such as SCFAs, LPS, and tryptophan derivatives interact with host receptors. Specifically, investigating the activation of G-protein coupled receptors (GPCRs) and the aryl hydrocarbon receptor (AhR) pathway will be crucial for understanding how the microbiome modulates neuroinflammation. To prove mechanistic causality, deeper validation utilizing germ-free animal models and “humanized” mouse models (colonized with patient microbiota) is required.

Ultimately, translating these findings into clinical practice requires a shift from empiric supplementation to precision microbiome medicine. As highlighted in this review, overcoming biological barriers such as mucosal colonization resistance and the “super-donor” phenomenon necessitates rigorous patient stratification based on baseline enterotypes and metabolic profiles. Future therapeutic strategies must prioritize mechanism-driven interventions—such as metabolic postbiotics or targeted prebiotics—that directly modulate specific neuroactive pathways (e.g., inhibition of Th1 differentiation, restoration of HDAC inhibition) rather than broadly altering taxonomic composition. Adopting such personalized and mechanistically grounded strategies represents a critical step toward translating the theoretical promise of the microbiota–gut–brain axis into tangible clinical benefits.

## Figures and Tables

**Table 1:** Summary of human studies investigating gut microbiota alterations in Alzheimer’s disease (AD).

n (Participants)	Increased Taxa	Decreased Taxa	Other Changes	Potential Mechanisms	Limitations	Reference
50 (25 AD, 25 HC)	Families: *Bacteroidaceae*, *Rikenellaceae*, *Gemellaceae* Genera: *Bacteroides*, *Blautia*, *Alistipes*, *Phascolarctobacterium*, *Bilophila*, *Gemella*	Families: *Ruminococcaceae*, *Bifidobacteriaceae*, *Peptostreptococcaceae*, *Turicibacteraceae* Genera: *Bifidobacterium*, *Dialister*, *Clostridium*, *Turicibacter*, *Adlercreutzia*	Reduced microbial diversity; distinct composition from age- and sex-matched controls	Dysbiosis-associated taxonomic shifts may correlate with AD pathology, although specific functional pathways were not assessed in this bacterial count analysis	Small sample size; cross-sectional design prevents causality assessment, functional pathways inferred rather than measured	[29]
83 (40 Amyloid+ CI, 33 Amyloid- CI, 10 HC)	Genera: Escherichia/Shigella, Spiecies: Pseudomonas aeruginosa	Species: Eubacterium rectale, Bacteroides fragilis	Increased abundance of pro-inflammatory taxa in amyloid-positive patients	Pro-inflammatory taxa may induce immune responses leading to Aβ deposition; loss of anti-inflammatory species	Cross-sectional design; focused on amyloid status rather than clinical AD diagnosis alone	[74]
86 (43 AD, 43 HC)	Phyla: *Actinobacteria* Genera: *Ruminococcus*	Phyla: *Bacteroidetes* Species: *Bacteroides fragilis*	Reduced diversity; specific alterations in *Ruminococcus* (mucus degraders)	*B. fragilis* depletion may increase gut permeability; *Ruminococcus* may impact gut health via mucus degradation	Relaively small sample size; potential confounding factors (e.g., long-term dietary habits) not fully controlled	[75]

Abbreviations: Aβ—amyloid beta; AD—Alzheimer’s disease; CI—cognitively impaired; HC—healthy controls. It should be noted that the reported taxonomic alterations are presented at different taxonomic levels (phylum, family, genus, or species), reflecting the heterogeneity of analytical approaches and reporting standards across the original studies.

**Table 2:** Summary of human studies investigating gut microbiota alterations in Parkinson’s disease (PD).

n (Participants)	Increased Taxa	Decreased Taxa	Other Changes	Potential Mechanisms	Limitations	Reference
144 (72 PD, 72 HC)	Families: *Enterobacteriaceae*, *Ruminococcaceae*	Families: *Prevotellaceae* (reduced by ~80%)	*Enterobacteriaceae* abundance correlated with the severity of postural instability and gait difficulty	Association with motor symptoms severity suggest a link between gut dysbiosis and PD clinical phenotype	Cross-sectional design; medication effects (e.g., COMT inhibitors) on microbiota not fully excluded	[37]
327 (197 PD, 130 HC)	Genera: *Akkermansia*, *Bifidobacterium*, *Lactobacillus*	Families: *Lachnospiraceae* Genera: *Blautia*	Altered pathways related to plant compound metabolism and xenobiotic degradation	Dysbiosis affects metabolic pathways; *Lachnospiraceae* depletion suggests reduced SCFA production	Potential confounding effects of PD medications (levodopa) and diet; cross-sectional design	[89]
175 (76 PD, 26 iRBD, 78 HC)	Genera: *Akkermansia*, *Prevotella*, *Anaerotruncus*, *Clostridium* XIVb, *Bacteroides*	Phyla: *Melainabacteria*	*Akkermansia* and *Prevotella* higher in PD with RBD; *Anaerotruncus* associated with non-motor symptoms	Gut microbiota composition correlates with specific non-motor symptoms (RBD), suggesting early involvement	Gut microbiota composition correlates with specific non-motor symptoms (RBD), suggesting early involvement; small sample size for the iRBD subgroup	[94]

Abbreviations: HC—healthy controls; iRBD—idiopathic rapid eye movement sleep behavior disorder; PD—Parkinson’s disease; RBD—rapid eye movement sleep behavior disorder; SCFA—short-chain fatty acids. It should be noted that the reported taxonomic alterations are presented at different taxonomic levels (phylum, family, genus, or species), reflecting heterogeneity in analytical approaches and reporting standards across the original studies.

**Table 3:** Summary of human and animal studies investigating gut microbiota alterations in amyotrophic lateral sclerosis (ALS).

n (Participants)	Increased Taxa	Decreased Taxa	Other Changes	Potential Mechanisms	Limitations	Reference
**Human Cohorts**						
100 (50 ALS, 50 HC)	Genera: *Escherichia*, *Enterobacter*	Other: Total Yeast	Clear dysbiosis was not evident; microbial profiles varied and overall complexity remained high.	Imbalance in pro/anti-inflammatory taxa may contribute to inflammation, though overall structure was not drastically disrupted	Sample size; lack of deep sequencing (focus on specific groups via qPCR)	[104]
57 (25 ALS, 32 HC)	Genera: Uncultured *Ruminococcaceae* (family-level)	None significantly altered	No significant difference in alpha/beta diversity	Dysbiosis was not evident; gut microbiota composition appeared stable	Strict patient selection (high functional status) might mask late-stage changes	[106]
185 (75 ALS, 110 HC)	Phyla: *Cyanobacteria*, *Bacteroidetes* Genera: *Bacteroides*, *Parasutterella*, *Lactococcu*	Phyla: *Firmicutes* Genera: *Faecalibacterium*, *Bifidobacterium*	Reduced α-diversity; correlation between specific taxa and plasma lipid metabolites	Impaired microbial homeostasis linked to lipid metabolism dysregulation; depletion of butyrate producers	Cross-sectional baseline data presented here (though study had longitudinal design); potential diet confounders.	[107]
139 (66 ALS, 61 HC, 12 NDC)	Genera: *Streptococcus*, *Escherichia*	Species: *Roseburia intestinalis*, *Eubacterium rectale* Genera: *Bilophila*, *Coprobacter*, *Eubacterium*	Overall diversity not significantly different; reduced total abundance of butyrate-producing species	Reduced butyrate production may exacerbate oxidative stress and neuroinflammation; pro-inflammatory taxa enrichment	Cross-sectional design; relatively small sample of neurodegenerative controls (NDC)	[98]
**Animal Models**						
Mouse Model (SOD1-G93A)	Genera: *Akkermansia muciniphila (AM)*, *Ruminococcus torques*, *Parabacteroides distasonis*, *Lactobacillus gasseri*, *Prevotella melaninogenica*	Not reported. Data focused on elevated taxa correlating with disease severity or amelioration	*AM* supplementation ameliorated symptoms; *R. torques* exacerbated them; nicotinamide levels reduced	Systemic nicotinamide depletion impairs motor neuron energetics; specific taxa modulate disease severity via metabolites	Animal model findings (caution required in translation); distinct vivarium-dependent microbiome	[102]

Abbreviations: ALS—amyotrophic lateral sclerosis; HC—healthy controls; NDC—neurodegenerative controls. Reported taxonomic alterations are presented at different taxonomic levels (phylum, family, genus, or species), reflecting heterogeneity in analytical approaches and reporting standards across studies. Animal model data are presented separately and are not directly comparable to human cohorts.

**Table 4:** Summary of metabolite-targeted intervention strategies, mechanisms, and clinical outcomes in neurodegenerative diseases.

Intervention Strategy	Target Metabolites and Molecular Mechanism	Clinical Outcome	Limitations	Reference
Dietary Modulation: (MIND Diet, Fiber)	Target: SCFA production (Butyrate) Mechanisms: Fiber fermentation inhibits HDACs: upregulation of tight junction proteins (Claudin-5), strengthens BBB integrity	AD: Reduced postmortem β-amyloid load (equivalent to ~4 years of younger age): lower incidence of AD	High variability in individual metabolic response: requires long-term adherence to show structural brain changes	[111,117]
Ologosaccharides (Sodium Oligomannate/GV-971)	Target: Phenylalanine, Isoleucine downregulation Mechanisms: Inhibition of amino acid-driven Th1 cell differentiation: blockade of peripheral immune infiltration into CNS	AD: Reversal of cognitive impairment in mild-to-moderate AD: remodeling of GM composition	Specificity to certain carbohydrate structures: dependency on baseline plasma amino acid levels	[109]
Metabolic Postbiotics (TUDCA + Sodium Phenylbutyrate)	Target: Bile Acids, Chaperones Mechanism: Mitigation of ER stress: prevention of mitochondrial dysfunction and neuronal apoptosis	ALS: Significant slowing of functional decline (ALSFRS-R scores) and extended survival in randomized trials	Gastrointestinal adverse events (diarrhea, nausea), high cost, taste palatability issues	[110]
Probiotics (*Lactobacillus*, *Bifidobacterium* **strains**)	Mechanism: Modulation of insulin signaling: suppression of NLRP3 inflammasome	PD: Improvement in metabolic status (insulin resistance) and constipation severity AD: Improved MMSE scores	Colonization Resistance: Stool shedding often reflects “washout” rather than mucosal engraftment; potential interference with native recovery post-antibiotics.	[114,115,118,119]
FMT	Target: Whole ecosystem restoration. Mechanism: Restoration of keystone species (*Lachnospiraceae*, *Ruminococcaceae*); eradication of SIBO.	PD: Reduction in motor (UPDRS) and non-motor symptoms, normalization of breath hydrogen levels (SIBO eradication)	Heterogeneity: Efficacy depends on “Super-Donor” status (richness of butyrate producers); lack of standardization	[116,120]

Abbreviations: AD—Alzheimer’s disease; ALS—amyotrophic lateral sclerosis; ALSFRS-R—ALS Functional Rating Scale-Revised; BBB—blood–brain barrier; CNS—central nervous system; ER—endoplasmic reticulum; FMT—fecal microbiota transplantation; GM—gut microbiota; HDAC—histone deacetylase; MIND—Mediterranean-DASH Intervention for Neurodegenerative Delay; MMSE—Mini-Mental State Examination; NLRP3—NLR family pyrin domain containing 3; PD—Parkinson’s disease; SCFA—short-chain fatty acids; SIBO—small intestinal bacterial overgrowth; Th1—T helper type 1 cells; TUDCA—tauroursodeoxycholic acid; UPDRS—Unified Parkinson’s Disease Rating Scale. Note: Mechanistic targets are primarily derived from preclinical models and in vitro studies, whereas reported outcomes reflect findings from human clinical trials.

## Data Availability

No new data were created or analyzed in this study. Data sharing is not applicable to this article.
